# Arthroscopic “Debridement and Implant Retention” With Local Administration of Exebacase (Lysin CF-301) Followed by Suppressive Tedizolid as Salvage Therapy in Elderly Patients for Relapsing Multidrug-Resistant *S. epidermidis* Prosthetic Knee Infection

**DOI:** 10.3389/fmed.2021.550853

**Published:** 2021-05-14

**Authors:** Tristan Ferry, Cécile Batailler, Aubin Souche, Cara Cassino, Christian Chidiac, Thomas Perpoint, Claire le Corvaisier, Jérôme Josse, Romain Gaillard, Julien Roger, Camille Kolenda, Sébastien Lustig, Frédéric Laurent, The Lyon BJI Study Group

**Affiliations:** ^1^Service des Maladies Infectieuses et Tropicales, Hôpital de la Croix-Rousse, Hospices Civils de Lyon, Lyon, France; ^2^Université Claude Bernard Lyon 1, Lyon, France; ^3^Centre Interrégional de Référence Pour la Prise en Charge des Infections Ostéo-Articulaires Complexes (CRIOAc Lyon), Hospices Civils de Lyon, Lyon, France; ^4^CIRI – Centre International de Recherche en Infectiologie, Inserm U1111, Université Claude Bernard Lyon 1, CNRS, UMR5308, Ecole Normale Supérieure de Lyon, Univ Lyon, Lyon, France; ^5^Service de Chirurgie Orthopédique, Hôpital de la Croix-Rousse, Hospices Civils de Lyon, Lyon, France; ^6^Institut des Agents Infectieux, Laboratoire de Bactériologie, Centre National de Référence des Staphylocoques, Hôpital de la Croix-Rousse, Hospices Civils de Lyon, Lyon, France; ^7^ContraFect Corporation, Yonkers, NY, United States; ^8^Pharmacie, Hôpital de la Croix-Rousse, Hospices Civils de Lyon, Lyon, France

**Keywords:** lysin, prosthetic-joint infection, tedizolid, staphylococci, *S. epidermidis*, bacteriophage

## Abstract

Exebacase, a recombinantly produced lysin has recently (i) reported proof-of-concept data from a phase II study in *S. aureus* bacteremia and (ii) demonstrated antibiofilm activity *in vitro* against *S. epidermidis*. In patients with relapsing multidrug-resistant (MDR) *S. epidermidis* prosthetic knee infection (PKI), the only surgical option is prosthesis exchange. In elderly patients who have undergone several revisions, prosthesis explantation could be associated with definitive loss of function and mortality. In our BJI reference regional center, arthroscopic debridement and implant retention with local administration of exebacase (LysinDAIR) followed by suppressive tedizolid as salvage therapy is proposed for elderly patients with recurrent MDR *S. epidermidis* PKI with no therapeutic option or therapeutic dead end (for whom revision or transfemoral amputation is not feasible and no other oral option is available). Each use was decided in agreement with the French health authority and in accordance with the local ethics committee. A written consent was obtained for each patient. Exebacase (75 mg/mL; 30 mL) was administered directly into the joint during arthroscopy. Four patients (79–89 years old) were treated with the LysinDAIR procedure. All had several previous prosthetic knee revisions without prosthesis loosening. Three had relapsing PKI despite suppressive antibiotics following open DAIR. Two had clinical signs of septic arthritis; the two others had sinus tract. After the LysinDAIR procedure, no adverse events occurred during arthroscopy; all patients received daptomycin 8 mg/kg and linezolid 600 mg bid (4–6 weeks) as primary therapy, followed by tedizolid 200 mg/day as suppressive therapy. At 6 months, recurrence of the sinus tract occurred in the two patients with sinus tract at baseline. After >1 year follow up, the clinical outcome was favorable in the last two patients with total disappearance of clinical signs of septic arthritis even if microbiological persistence was detected in one of them. Exebacase has the potential to be used in patients with staphylococci PKI during arthroscopic DAIR as salvage therapy to improve the efficacy of suppressive antibiotics and to prevent major loss of function.

## Introduction

Prosthetic joint infection (PJI) is the most dramatic complication after joint arthroplasty. *S. aureus* and coagulase-negative staphylococci are frequently involved in patients with PJI (1). These bacteria could be involved in recurrence as they can produce biofilm and persist at the implant surface (2). In patients with acute staphylococci PJI, the recommended medico-surgical strategy is to perform an open debridement antibiotics and implant retention (DAIR) with exchange of the mobile polyethylene part, followed by an antibiotic regimen that includes rifampin, which demonstrates antibiofilm activity (3–5). Arthroscopic DAIR is contraindicated in patients with PJI as (i) the risk of relapse is particularly high if the polyethylene part cannot be changed, likely because such a plastic surface promotes biofilm formation, and (ii) the reduction of the bacterial load is significantly lower in comparison with open DAIR (6–8). In patients with chronic PJI, the recommended strategy is to exchange the prosthesis, in a one- or two-stage procedure, to mechanically eradicate the biofilm (3–5). In patients with relapsing or chronic staphylococci PJI, prosthesis explanation is sometimes not feasible, especially for the knee location in elderly patients with multiple comorbidities for whom explantation could be associated with a dramatic loss of function, reduction of the bone stock, fracture or peroperative death. Indeed, explantation without reimplantation, also called resection arthroplasty or the Girldestone procedure, is possible for the hip but not for the knee. Open DAIR is sometimes proposed for patients with relapsing or chronic staphylococci PJI, but as the risk of relapse is particularly high due to the bacterial persistence in biofilm, these patients are candidates for suppressive antibiotic treatment (SAT) (3–5). SAT consists of daily oral intake of active antibiotic to suppress the infection, i.e., to alleviate the symptoms and prevent the progression of the infection without hope for eradication. In cohort studies, the outcome is favorable in 30–70% of patients, depending on the patient profile, the pathogen involved, the drug used, and the duration of follow up (9–14). Doxycycline, cotrimoxazole, or cephalexin are the most frequently used drugs for SAT in staphylococcal PJI (9, 11, 12, 14). In patients with multidrug-resistant (MDR) coagulase negative staphylococci PJI, linezolid is frequently the only oral active drug. However, its use is associated with a significant toxicity when prescribed for >28 days (15, 16). In this context, the use of new adjuvant therapies is of great interest and may improve the stabilization of medical conditions of patient with PJI.

Lysins are cell wall hydrolase enzymes produced by bacteriophage during their lytic circle (17). As recombinantly produced proteins, lysins trigger rapid peptidoglycan hydrolysis, osmotic lysis, and cell death upon contact with bacteria. In contrast, antibiotic-mediated killing may require up to several hours. Lysin exebacase (CF-301) is an anti-staphylococcal lysin with potent bactericidal activity against *S. aureus* and additionally against coagulase-negative staphylococci. Exebacase is, furthermore, shown to disrupt mature biofilms formed by a wide range of methicillin-sensitive and -resistant *S. aureus* (MSSA) isolates as well as coagulase-negative staphylococci (18). The ability of exebacase to both eradicate biofilm biomass and kill bacteria in biofilm is demonstrated on a variety of surfaces, including catheters (19). Recently, a phase 2 superiority design clinical study, performed in adult patients to evaluate safety, tolerability, efficacy, and PK of exebacase when used in addition to standard-of-care (SoC) antibiotics for the treatment of *S.aureus* bacteremia, including endocarditis, revealed an improved outcome in patients receiving exebacase (20).

In France, 10 years ago, the ministry of health implemented a network of nine reference regional centers called “CRIOAc” to promote the research and management in the field of complex bone and joint infections. In our center, various strategies have been developed to try to control chronic infections in patients with PJI for whom prosthesis revision is not feasible (21). Some patients have been treated with therapeutic GMP/GMP-like-produced bacteriophages targeting *P. aeruginosa* or *S. aureus*. Unfortunately, no therapeutic bacteriophages active against *S. epidermidis* are available for compassionate treatment in France (22, 23), whereas we have had patients with relapsing MDR *S. epidermidis* prosthetic knee infection (PKI) who experienced iterative relapses, sometimes under SAT, after open DAIR. For such patients at a therapeutic dead end, we proposed arthroscopic DAIR with local administration of exebacase (LysinDAIR procedure) based on its antibiofilm activity against *S. epidermidis*, as compassionate treatment, followed by suppressive tedizolid as salvage therapy.

## Methods

Based on the use of exebacase in France for the phase 2 study in bacteremia, individual requests were done for successive patients to the French Health Authority, *Agence Nationale de Sécurité du Médicament et des Produits de Santé* (ANSM), to gain approval to perform LysinDAIR. In accordance with the local ethics committee, each case was discussed individually during multidisciplinary meetings in our CRIOAc center and then with ANSM to be sure that no other options associated with considerable loss of function or risk of death could be proposed. Exebacase MICs were evaluated for the *S. epidermidis* PJI clinical strains isolated from patient samples, using the Clinical and Laboratory Standards Institute (CLSI)-approved medium CAMHB-HSD composed of cation-adjusted Mueller-Hinton broth (CAMHB, BD BBL^TM^) supplemented with 25% horse serum (Sigma-Aldrich) and 0.5 mM DTT (Dithiothreitol, Sigma-Aldrich) (24). MICs were determined after 18 h of incubation at 37°C as previously described (25). After authorization for compassionate use of exebacase from health authorities, a written consent was obtained for each patient, and the surgery planned. Conventional arthroscopy was performed, using anteromedial and anterolateral entry points, allowing sampling for bacterial cultures (joint fluid, synovial, and bone tissue) and washing of the joint with saline. In patients with a sinus tract, its resection (fistulectomy) was systemically performed. After drainage of the arthroscopic liquid, 30–50 cc of a solution containing Exebecase diluted in glucose 5% (final concentration 0.075mg/mL) were injected. Then, entry points were closed to be waterproof.

## Results

**Figure 1:**
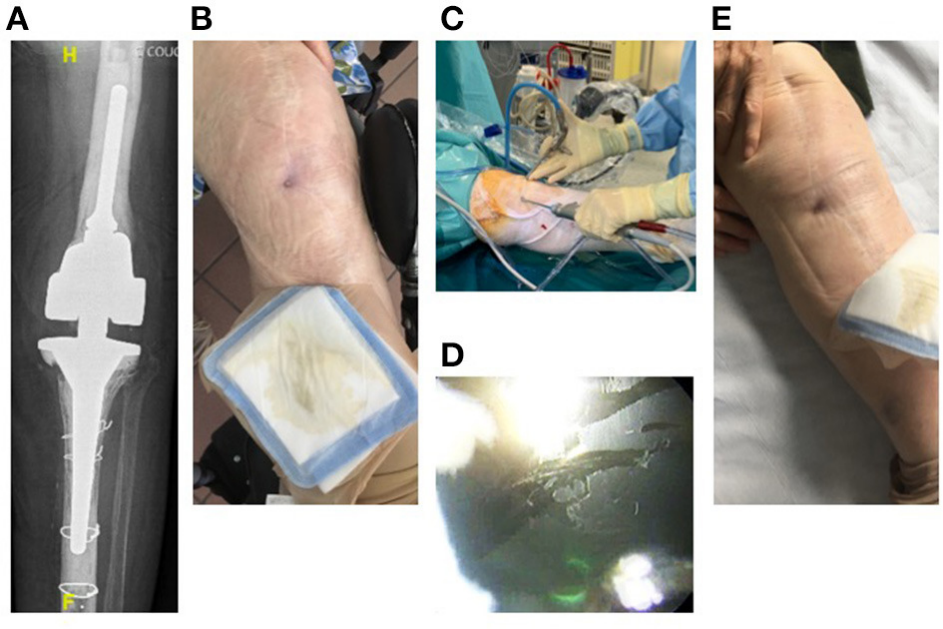
Patient 1 was an 89-year-old woman with past history of recurrent lymphoma, splenectomy, and iterative prosthetic left knee revisions due to relapsing MDR *S. epidermidis* infection (04/02/2016). She had large constrained cemented prosthesis without loosening **(A)** and sinus tract **(B)**. She experienced a relapse under SAT (pristinamycin plus doxycyclin) following open DAIR 1 year ago. She was treated according to the LysinDAIR procedure **(C)**. The biofilm was visible at the surface of the implant during the DAIR procedure [08/11/2018; **(D)**]. She received daptomycin intravenously and linezolid orally, followed by tedizolid as SAT. At 6 months, still receiving tedizolid therapy, a new discharge occurred through the sinus tract **(E)**.

**Figure 2:**
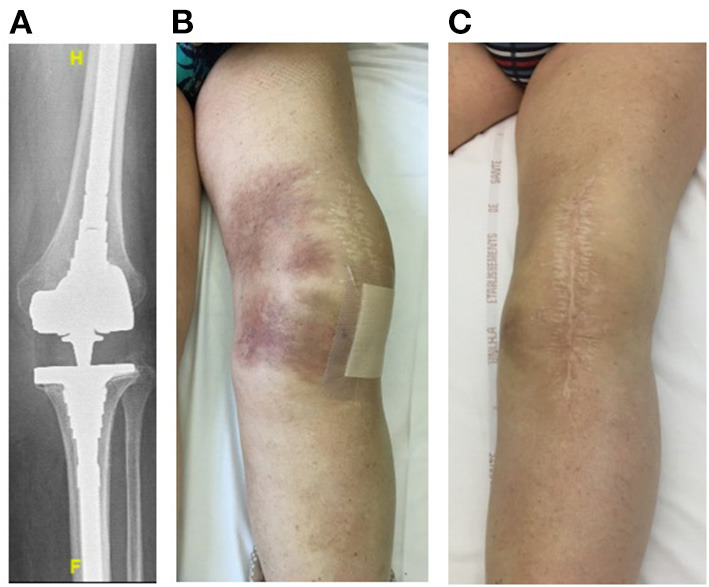
Patient 2 was a 79-year-old man with history of severe ankylosing spondylitis under corticosteroids who presented a chronic left PKI due to *S. hominis* that was treated with a one-stage exchange. A postoperative infection occurred due to MDR *S. epidermidis* (02/12/3013) treated with open DAIR and SAT (minocycline followed by cotrimoxazole due to occurrence of a clinical relapse under minocycline therapy; 10/11/2013). He had a cementless revision prosthesis with long stem with no loosening **(A)** and clinical signs of septic arthritis (large joint effusion, pain during mobilization, skin inflammation without sinus tract) **(B)** and *S. epidermidis* grew from joint puncture (9/13/2018). He was treated according to the LysinDAIR procedure (08/11/2018), and a septic collection communicating with the joint was drained. He received daptomycin intravenously and linezolid orally and experienced eosinophilic pneumonia attributed to daptomycin and diarrhea attributed to linezolid. Then, tedizolid was prescribed as SAT, and the outcome was favorable with disappearance of the clinical signs of septic arthritis **(C)**. At 12 months, as a mild joint effusion persisted, a joint puncture was performed, and surprisingly, *S. epidermidis* was still present in culture. At the time of writing (16 months of follow-up after the LysinDAIR procedure), the clinical outcome was still favorable under tedizolid therapy, and the patient was able to resume golf.

**Figure 3:**
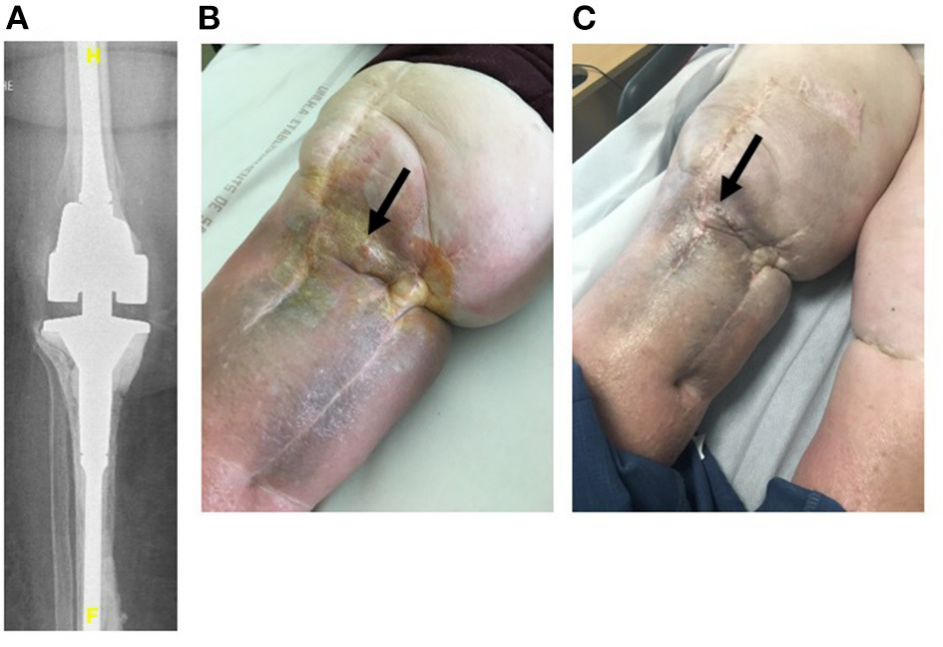
Patient 3 was an 88-year-old woman with a current history of active chronic myeloid leukemia under imatinib therapy requiring iterative blood perfusions. She also had chronic kidney disease with indication of dialysis that the patient refused. She also had chronic lymphoedema. She had a right-cemented revision prosthesis **(A)** following a two-stage exchange for PKI due to MDR *S. epidermidis* (04/02/2011). Unfortunately, *S. epidermidis* persistence was diagnosed (6/7/2018), and a sinus tract occurred **(B)**. Cotrimoxazole as SAT was contraindicated due to the kidney disease. The patient received intermittent antimicrobial therapy with pristinamycin when skin and soft tissue inflammation occurred around the sinus tract **(B)**. She was treated according to the LysinDAIR procedure (10/01/2019). She received daptomycin intravenously and linezolid orally and experienced severe thrombopenia (30 G/L) leading to discontinuation of linezolid and a switch to tedizolid. Unfortunately, at 6 months, under tedizolid therapy, a new discharge occurred trough the sinus tract **(C)**.

**Figure 4:**
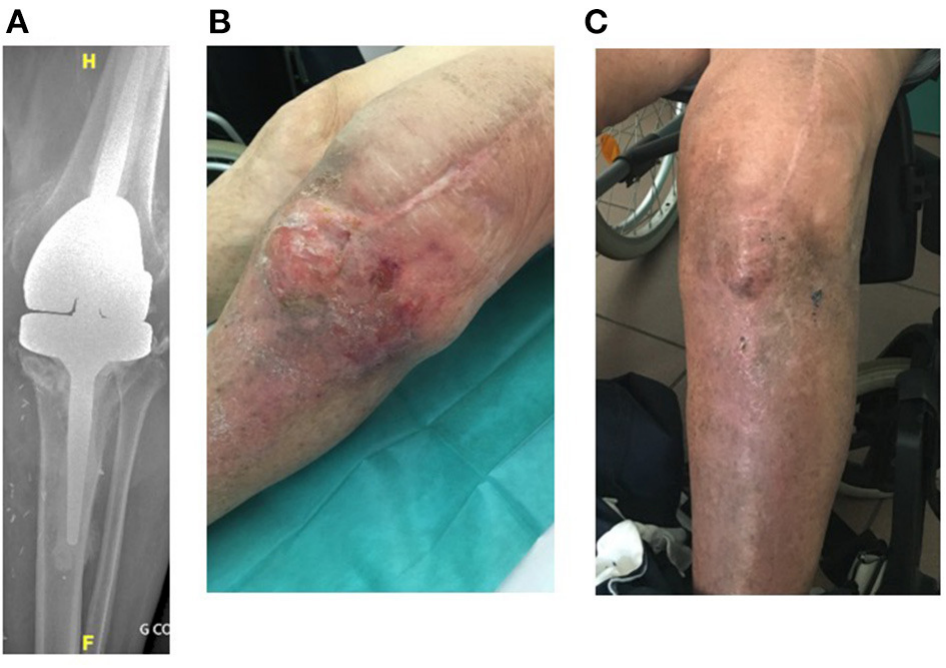
Patient 4 was an 83-year-old man with a history of severe cardiomyopathy requiring anticoagulation, dyslipidemia, and diabetes. He had a left-cemented revision prosthesis **(A)** following a two-stage exchange for PKI due to *Streptococcus* spp. He experienced postoperative chronic septic arthritis due to *S. epidermidis* (5/23/2018), and he was treated by open DAIR, skin and soft tissue flap, and SAT (clindamycin plus levofloxacin followed by clindamycin plus cotrimoxazole). Under SAT, the patient experienced a relapse of the septic arthritis **(B)** due to MDR *S. epidermidis* (19/07/2018). He was treated according to the LysinDAIR procedure (10/01/2019) and received daptomycin intravenously and linezolid orally. He developed eosinophilic pneumonia attributed to daptomycin before the switch to tedizolid. During the follow-up, the clinical signs of septic arthritis totally disappeared. At the time of writing (14 months of follow-up after the LysinDAIR procedure), the clinical outcome was still favorable under tedizolid **(C)**.

Four patients (79–89 years old) with significant comorbidities were treated with the LysinDAIR procedure as salvage therapy (Figures 1–**4**). All had undergone several previous prosthetic knee revisions without prosthesis loosening (Figures 1A–**4A**). Three had relapsing PKI despite suppressive antibiotics following open DAIR. Two had clinical signs of septic arthritis (Figures 2B, **4B**); the two others had sinus tract (Figures 1B, 3B). All patients were infected only by *S. epidermidis* that expressed different drug susceptibilities over time, likely due to small colony variant phenotype and/or co-infection with different strains of *S. epidermidis* (Table 1). Despite the fact that past isolates were no more available for further drug susceptibility testing, based on the previous and current antimicrobial susceptibility test and patients' comorbidities, tedizolid was the only drug candidate to be used as potential SAT. Exebacase MIC values are detailed in Table 1. No adverse events occurred during arthroscopy (Figure 1C). The biofilm was clearly visible during arthroscopy for one patient (Figure 1D). All patients received intravenous daptomycin (8 mg/kg) immediately after arthroscopy and oral linezolid (600 mg bid) for 4–6 weeks, followed by oral tedizolid 200 mg/day (one pill) as suppressive therapy. During the treatment, two patients developed eosinophilic pneumonia attributed to daptomycin, one patient experienced diarrhea under linezolid therapy (without *C. difficile* infection), and another patient developed worsening of a previous thrombopenia under linezolid therapy. No adverse event was noticed under tedizolid treatment, in particular, neither myelotoxicity nor neurotoxicity. At 6 months, under tedizolid therapy, recurrence of the sinus tract occurred in the two patients with sinus tract at baseline (Figure 1E, Figure 3C). After >1 year of follow up (respectively, 14 and 16 months), the clinical outcome was decidedly favorable for the two last patients with complete disappearance of clinical signs of septic arthritis (Figures 2C, 4C). As a mild joint effusion persisted in one of them (patient 2), a joint puncture was performed. Surprisingly, it revealed the persistence of the *S. epidermidis*, that remained susceptible to linezolid and tedizolid.

**Table 1:** Antibiograms and exebacase MIC of the patients' *S. epidermidis* isolates.

**Patient**	**PJI episode**	**Sampling date**	**Exebacase MIC (mg/L)**	**Antbiotics**												
				**OXA**	**K**	**GM**	**NN**	**E**	**L**	**TE**	**OFX**	**SXT**	**FA**	**RA**	**VA**	**LZD**
Patient 1	Previous episode	04/02/2016	NT	R	R	R	R	R	R	I	R	R	R	R	S	S
	Joint puncture before surgery	17/11/2017	1	R	R	R	R	R	R	I	R	R	R	R	S	S
	At the time of surgery	08/11/2018	2	R	R	R	R	R	R	I	R	R	R	R	S	S
Patient 2	Previous episode	02/12/2013	NT	R	S	S	S	R	R	S	S	R	R	R	S	S
	Previous episode	11/10/2013	NT	S	R	R	R	R	R	S	R	R	R	R	S	S
	Joint puncture before surgery	13/09/2018	0.125	S	S	S	S	S	S	S	S	S	S	NT	S	S
	At the time of surgery^*^	08/11/2018	NA	NA	NA	NA	NA	NA	NA	NA	NA	NA	NA	NA	NA	NA
Patient 3	Previous episode	04/02/2011	NT	R	R	R	R	R	R	R	R	S	R	NT	S	S
	Joint puncture before surgery	07/06/2018	0.25	S	R	R	R	R	R	R	R	S	R	R	S	S
	At the time of surgery	10/01/2019	2	S	R	R	R	R	R	R	R	I	R	R	S	S
Patient 4	Previous episode	23/05/2018	NT	S	S	S	S	R	S	S	S	S	R	S	S	S
	Joint puncture before surgery	19/07/2018	ID	S	S	S	S	R	S	S	S	S	S	S	S	S
	At the time of surgery	10/01/2019	NT	R	R	R	R	R	R	S	R	R	R	S	S	S

*OXA, oxacillin; K, kanamycin; GM, gentamicin; NN, tobramycin; E, erythromycin; L, Lincomycin; TE, tetracycline; OFX, ofloxacin; SXT, cotrimoxazole; FA, fusidic acid; RA, rifampin; VA, vancomycin; LZD, linezolid; NA, strain not available; ID, impossible to determine as no bacterial growth was observed in the specific media (CaMHB-HSD composed of cation adjusted Mueller-Hinton broth) used for MIC determination; NT, not tested; R (in red), resistant; I (in orange), intermediate; S (in green), susceptible*.

* *The patient had surgery under cotrimoxazole therapy that probably inhibited the growth of S. epidermidis from peroperative samples*.

## Discussion

We report the compassionate use of exebacase administered locally during arthroscopy in four patients with relapsing MDR *S. epidermidis* PKI. This use is based on the crucial need for adjuvant therapeutic innovation for the management of patients with PKI, especially if MDR staphylococci are involved and if prosthesis revision is not feasible. Indeed, explantation without reimplantation (resection arthroplasty, also called the Girdlestone procedure) is, in theory, not acceptable for the knee location, whereas it is a possible option in patients with chronic prosthetic hip infection. Goldman et al. recently reports the functional outcome of patients with definitive resection arthroplasty of the knee, and even if this procedure facilitated the cure of the infection, all patients had residual pain, instability, and needed hinged orthosis with limited mobility. Arthrodesis using a silver-coated Arthrodesis implant or performing transfemoral amputation are other surgical options (26, 27). The latter option is associated to a catastrophic outcome and needs to be absolutely avoided (27).

SAT is seen as an alternative strategy for cases of PJI in which prosthesis explantation, i.e., biofilm eradication, could not be performed. SAT consists of the indefinite administration of antibiotics, and its goal is to control the infection, i.e., to reduce and ideally make disappear the clinical symptoms and slow down the occurrence of mechanical complications, such as prosthesis loosening. SAT is an infrequent therapeutic option but could be of importance in the elderly (9–14). The Spanish Society of Infectious Diseases and Clinical Microbiology (SEIMC) states that the following conditions need to be met for the indication of SAT: (i) identification of the microorganism causing the infection, (ii) availability of oral antibiotics that are not toxic when administered over long periods of time, and (iii) possibility of close follow-up of the patient. This group states that it is reasonable to think that reducing the bacterial inoculum and debriding the infected tissues may favor the success of SAT and that a new debridement would allow the taking of good-quality tissue samples for culture before starting SAT (5). It is not known if the use of antibiofilm agents just after surgery and before prescribing SAT could facilitate the rate of success of SAT. The benefit of using rifampin at the initial phase of treatment of SAT is not clear as discussed in the Infectious Diseases Society (IDSA) guidelines that proposed to use cotrimoxazole or minocycline or doxycycline as SAT in patients with MDR staphylococci PJI (3).

Here, in this context, patients experiencing relapsing PKI despite previous prosthesis revision and open DAIR followed by SAT were selected for an innovative DAIR approach in including local phage therapy. We proposed arthroscopic DAIR to limit the risk of perioperative complications and the risk of superinfection. Indeed, arthroscopic DAIR is usually contraindicated in patients with PKI, but in the present cases, it facilitates the use of an antibiofilm agent that could be injected during the DAIR procedure. Thus, it is easy to inject into the joint a solution during arthroscopy, and the tightness of the joint is considerably better after arthroscopy in comparison with arthrotomy with less leakage of joint fluid through the scar. The use of exebacase is based on the fact that it has demonstrated an *in vitro* antibiofilm activity on *S. aureus* and against *S. epidermidis* strains in various models, such as *in vitro* models on polystyrene, glass, surgical mesh, and catheter (19). Moreover, it demonstrated *in vitro* synergy with a broad range of antibiotics against both methicillin-susceptible and -resistant *S. aureus* (18). Exebacase also is shown to be more active in combination with daptomycin than daptomycin or exebacase alone to treat methicillin-resistant *S. aureus* acute osteomyelitis in rats (28). Notably, the exebacase MIC values reported for isolates in this study were within the range of 0.125–2 μg/mL previously reported for *S. epidermidis* (18).

The choice of tedizolid for oral SAT is based on the fact that this drug has a strong potential in patients with PJI as several case reports and case series report its safe prolonged use (29, 30). Moreover, this drug remains active in MDR staphylococci and could have potential activity against persisters (31). However, PJI is an off-label use of tedizolid, and this antibiotic is a costly option for SAT as a one-year supply of this drug is approximately $127,000 in the United States and €75,000 in France (16).

As the selected patients here already experienced a relapse despite open DAIR and SAT, the rate of expected success, if exebacase had no effect on the biofilm, was close to zero. Even though we observed a relapse in the two patients with sinus tract, the impressive significant clinical outcome in the two other patients make the LysinDAIR procedure a potential innovative approach that need to be investigated. In the study of Prendki et al., (10)experiencing a sinus tract before the implementation of SAT was a risk factor for failure, but no surgery was performed in most of these patients from this study, and other studies (9, 11–14), as per published guidelines in the field (3–5), did not suggest that sinus tract should contraindicate the performance of DAIR followed by SAT in patients with chronic PJI.

Based on the present data, exebacase showed the potential to be used as salvage therapy administered during arthroscopic DAIR procedure in patients with staphylococci PKI, to improve the efficacy of SAT and to avoid considerable loss of function. The observed initial clinical response in all patients and sustained clinical responses in two of the four suggests that the use of exebacase intra-articularly for PJI warrents further study to refine dosing and frequency of administration. The fact that exebacase was well-tolerated with no adverse events related to the arthroscopic administration and no events of hypersensitivity to the drug is encouraging, and this, together with the early signals of clinical response, warrant further investigation to refine dosing in a Phase 1 B design clinical study.

## Data Availability Statement

The original contributions presented in the study are included in the article/supplementary material, further inquiries can be directed to the corresponding author.

## Ethics Statement

Written, informed consent was obtained from each patients for the publication of any potentially identifiable images or data included in this article.

## Lyon BJI Study Group

**Coordinator**: Tristan Ferry; **Infectious Diseases Specialists**: Tristan Ferry, Florent Valour, Thomas Perpoint, Patrick Miailhes, Florence Ader, Sandrine Roux, Agathe Becker, Claire Triffault-Fillit, Anne Conrad, Cécile Pouderoux, Nicolas Benech, Pierre Chauvelot, Marielle Perry, Fatiha Daoud, Johanna Lippman, Evelyne Braun, Christian Chidiac; **Surgeons**: Sébastien Lustig, Elvire Servien, Cécile Batailler, Stanislas Gunst, Axel Schimdt, Matthieu Malatray, Eliott Sappey-Marinier, Michel-Henry Fessy, Anthony Viste, Jean-Luc Besse, Philippe Chaudier, Lucie Louboutin, Quentin Ode, Adrien Van Haecke, Marcelle Mercier, Vincent Belgaid, Arnaud Walch, Sébastien Martres, Franck Trouillet, Cédric Barrey, Ali Mojallal, Sophie Brosset, Camille Hanriat, Hélène Person, Nicolas Sigaux, Philippe Céruse, Carine Fuchsmann; **Anesthesiologists**: Frédéric Aubrun, Mikhail Dziadzko, Caroline Macabéo; **Microbiologists**: Frederic Laurent, Laetitia Beraut, Tiphaine Roussel-Gaillard, Céline Dupieux, Camille Kolenda, Jérôme Josse; **Imaging**: Fabien Craighero, Loic Boussel, Jean-Baptiste Pialat, Isabelle Morelec; **PK/PD Specialists**: Michel Tod, Marie-Claude Gagnieu, Sylvain Goutelle; **Clinical Research Assistant and Database Manager**: Eugénie Mabrut.

## Author Contributions

TF managed all the patients, directly interacted with the French Health authority, and wrote the manuscript. CB, SL, RG, and JR performed the arthroscopic lavage. FL, JJ, AS, and CK performed bacteriological experiments. All authors participated to the literature review and the improvement of the manuscript.

## Conflict of Interest

CCa is employed by the company Contrafect. TF received honorarium as speaker from Contrafect to present these results to the company members. The Hospices Civils de Lyon - Institut des Agents Infectieux received financial support for a research project aimed at evaluating *in vitro* Lysin CF-301 activity on a collection of clinical strains. The remaining authors declare that the research was conducted in the absence of any commercial or financial relationships that could be construed as a potential conflict of interest.
